# Development and Performance Evaluation of Solid–Liquid Two-Component Coatings for Airport Cement Pavement Focused on Texture Reconstruction

**DOI:** 10.3390/ma18225171

**Published:** 2025-11-14

**Authors:** Ming Wang, Shuaituan Tian, Lingyun Zou, Mingchen Li, Jinlin Huang, Junyan Zhi

**Affiliations:** 1 Transportation Science and Engineering College, Civil Aviation University of China, Tianjin 300300, China; m_wang@cauc.edu.cn (M.W.); 231440245@cauc.edu.cn (J.H.); 18826133408@163.com (J.Z.); 2 Civil Aviation Research Base (Beijing) Co., Ltd., Beijing 100621, China; 17312266273@163.com; 3 China Airport Construction Group Co., Ltd., Beijing 100621, China; 4 School of Infrastructure Engineering, Dalian University of Technology, Dalian 116024, China; mcli@dlut.edu.cn

**Keywords:** road engineering, airport pavement, composite coatings, solid–liquid two-component, texture characterization, skid resistance, Interfacial strength, frost resistance

## Abstract

Pavement texture is a crucial factor influencing both skid resistance and durability. This study aims to investigate the impact of texture reconstruction on pavement performance, which holds significant scientific value for enhancing road safety and durability. The research focuses on the reconstruction of airport cement pavement textures through the design of seven solid–liquid, two-component coating formulations, comprising three types of coatings: emulsion coating (P), waterborne epoxy coating (E), and water-based coating (W). Laser texture scanning technology was employed to identify the texture characteristics, which, combined with the British pendulum test, enabled a comprehensive analysis of skid resistance. Additionally, the coating–concrete interfacial strength and frost resistance were evaluated through pull-out tests, flexural strength tests, and freeze–thaw cycle tests. The results demonstrated that, compared to uncoated concrete, the mean profile depth (MPD) of the P, E, and W coatings increased by 43.4%, 34.7%, and 21.6%, respectively. Furthermore, the peak band of the slope spectrum density (SSD) shifted from a range greater than 1 mm to approximately 0.5 mm following coating application. The British pendulum number (BPN) increased by 25%, 20%, and 15% for the P, E and W coatings, demonstrating a strong correlation with MPD (R^2^ = 0.95). These results indicate that the coated surface texture exhibits superior properties, which explain the enhanced slip resistance from a textural perspective. Moreover, the interfacial strength between the coating and concrete initially increased and then decreased with increasing coating thickness. In comparison, the interfacial bonding strength of the E coating was significantly higher than that of the P and W coatings. Furthermore, compared to the P and W coatings, the flexural bond strength of the E coating increased by 7% and 74%, respectively. After undergoing the freeze–thaw cycle, the E coating exhibited the best freeze resistance, while the W coating exhibited the poorest performance. In summary, the P coating excelled in texture reconstruction, while the E coating provided superior bonding and freeze resistance. This paper presents a novel approach to the development of coating materials for use on airport pavements.

## 1. Introduction

The skid resistance and protective performance of pavement surfaces directly influence the operational safety of aircraft. However, surface defects, such as skid resistance failure and freeze–thaw damage, are commonly observed in in-service airport pavements [1]. Given the need for construction to proceed without interrupting flight operations, there is an urgent demand for airports to enhance the skid resistance and protective performance of their pavements rapidly. The coefficient of friction of a pavement is contingent upon its surface texture structure, and pavement texture reconstruction has been shown to improve skid resistance [2]. Current repair techniques, such as slab replacement and asphalt overlay, are characterized by high costs, complex procedures, and suboptimal post-repair durability. Therefore, it is imperative to explore innovative and rapid pavement texture reconstruction technologies.

Concrete repair coating technologies have attracted considerable attention in recent years. Studies have explored various approaches, including surface treatments to enhance durability [3], the development of innovative protective coatings such as inhibitor-loaded intelligent coatings for steel [4] and asphalt polyurethane for concrete [5], and the application of silane and superhydrophobic coatings for airport pavements [6,7,8,9]. Furthermore, research has shown that both material composition and surface texture are critical for performance. For instance, modified polyurea elastomers and engineered cement composites have been proven to significantly improve abrasion resistance and skid resistance [10,11], a benefit also derived from positive texture characteristics and curved texturing techniques, which enhance friction by promoting rubber hysteresis and facilitating drainage [12,13,14,15]. Despite the well-established importance of pavement texture, a significant scientific and practical challenge persists: the absence of rapid, cost-effective, and durable technologies specifically designed for reconstructing surface texture on airport cement pavements [16]. Although existing research on concrete durability is extensive. Recent studies on airport pavement concrete have focused on enhancing its durability and performance using ultra-fine supplementary cementitious materials, advanced material design, and the incorporation of fibers with surface treatment, representing core pathways for material improvement [17,18,19]. Moreover, some studies have centered on the durability of concrete paving at airports under the dual impact of freeze–thaw cycles and deicing salts [20,21]. Furthermore, surface coatings for protection are well-established. Shen et al. focused on developing and evaluating advanced functional surface layers for pavements, with a shared emphasis on quantifying and ensuring their skid resistance performance through experimental characterization and performance measurement [22,23,24]. Coating-combined application for the functional reconstruction of texture to enhance skid resistance, while maintaining or improving interfacial and freeze–thaw properties, remains underexplored.

Hence, the primary objective of this study is to develop and systematically evaluate a series of novel solid–liquid two-component coatings designed specifically for this purpose. The specific objectives include formulating three types of coatings (emulsion P, waterborne epoxy E, and water W) with varying solid–liquid ratios; quantitatively characterizing their effectiveness in reconstructing surface texture using laser scanning; and evaluating their functional performance in terms of skid resistance (BPN), coating–concrete interfacial strength (pull-out and flexural bond) [25], and freeze–thaw durability. In addition, the study recommended optimal coating formulations based on a comprehensive performance analysis.

## 2. Materials and Methods

### 2.1. Coating Material Composition and Preparation

The composition of the coating materials is found to have a significant impact on their performance characteristics. The preparation method is critical for ensuring that the designed components are homogeneously integrated and that the intended properties are fully achieved in the final coating. It is imperative to provide a comprehensive account of the composition and preparation process to define the coating system and thereby facilitate a comprehensive understanding of its subsequent performance.

#### 2.1.1. Material Composition

**Figure 1:**
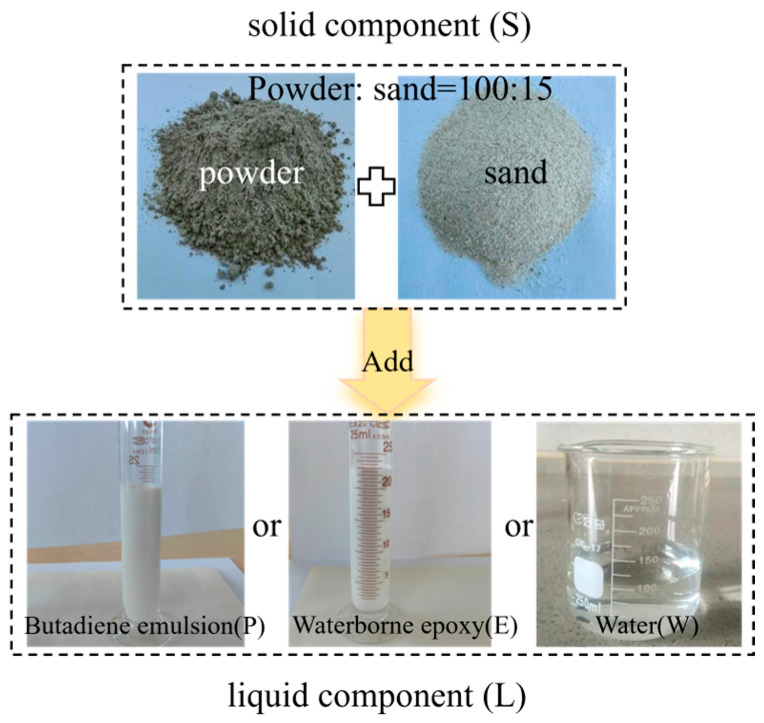
The material’s appearance required for coating preparation.

The coating material prepared in this study consists of a solid–liquid two-component system. The solid component (denoted as S) is a self-prepared powder that contains nano-calcium carbonate and nano-titanium dioxide. This powder, primarily composed of portland cement (525), sulfate aluminate cement (525 or 725), and early-strength agents, is mixed with 100-mesh ultrafine quartz sand at a fixed ratio of powder to sand (100:15). The liquid component (denoted as L) consists of self-prepared emulsions, including high-purity styrene-butadiene rubber latex with a solid content greater than 45%, high-purity acrylic emulsion, and silica sol, along with waterborne epoxy and pure water. Waterborne epoxy was included due to its reputation for high cohesive strength and superior chemical resistance [26]. Among these, the curing agent added to the waterborne epoxy resin is a water-soluble epoxy curing agent. Both the waterborne epoxy resin and the waterborne epoxy curing agent were purchased from Shenzhen Jitian Chemical Co., Ltd. (Shenzhen, China). During the preparation process, the liquid components are combined with the solid component in varying proportions to achieve the desired coating materials. For clarity in subsequent research and descriptions, the coatings prepared with emulsions, waterborne epoxy, and pure water are labeled as P, E, and W, respectively. The appearance of the individual coating components is shown in Figure 1.

#### 2.1.2. Preparation Method

**Figure 2:**
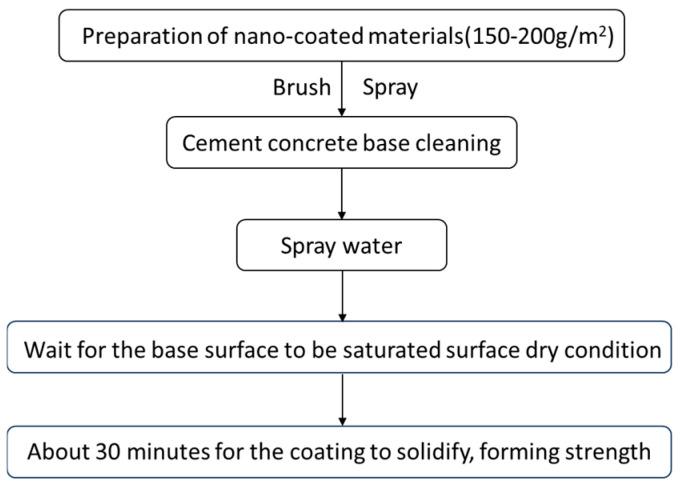
Coating application process flow.

The preparation of the coating material consists of the following steps. First, the solid and liquid components were precisely weighed according to the predetermined proportions. The liquid components were then diluted to the specified volume ratio and transferred to a container fitted with a stirring device. The mixture was stirred to ensure uniformity before the gradual addition of the solid components. Second, all components were thoroughly integrated, and stirring was continued for an additional 3–5 min to obtain a homogeneous final nano-coating material. The coating was then applied to the concrete surface using either brushing or rolling techniques, with an application rate of approximately 150–200 g/m^2^. The main process flow for the coating application is illustrated in Figure 2.

The ratio of solid to liquid components (i.e., solid–liquid ratio) directly influences the technical performance and cost of the coating material. According to the Cementitious Self-Leveling Compound for Floor (JC/T 985-2017) [27], fluidity tests were conducted on coatings with varying solid–liquid ratios. The flowability value was specified to be no less than 90 mm, based on the experience of the project team and the requirements of large-scale spraying construction on-site. The selection of seven coating formulations was made by the flowability test results for each formulation and economic factors. The uncoated condition is denoted by N. For the specific coating formulations and their identifiers, refer to Table 1.

### 2.2. Coating Performance Evaluation Methods

This section presents a comprehensive evaluation of coating performance by outlining the primary test methods employed to assess its characteristics.

#### 2.2.1. Laser Texture Scanning Test

**Figure 3:**
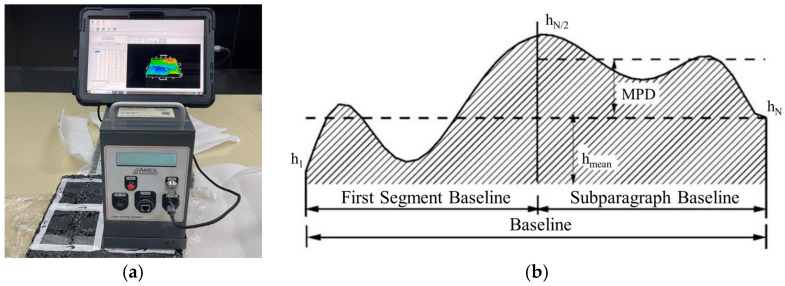
Texture characterization: (**a**) Experimental setup; (**b**) Principle of MPD calculation.

The AMES laser texture scanner was utilized to assess the multidimensional metrics of the surface texture morphology of the coatings, as shown in Figure 3a. The mean profile depth (MPD) and slope spectral density (SSD) were measured to characterize the surface texture. The meaning of the MPD metric is illustrated in Figure 3b. The mathematical significance of SSD is the root mean square value of the texture slope distribution at the sampled coordinates, calculated according to Equation (1) [28].

(1) $$SSD_{\lambda }=\sqrt{\frac{1}{N}\sum_{i=1}^{N}{\left(\frac{z_{i+1}+z_{i}}{\Delta x}\right)}^{2}}$$

*SSD_λ_* is the slope spectral density at the structural wavelength *λ*; *N* is the number of coordinates in the baseline; *z_i_* is the elevation value at coordinate *i*; and Δ*x* is the horizontal distance between coordinates.

#### 2.2.2. British Pendulum Test

In accordance with the Field Test Methods of Highway Subgrade and Pavement (JTG 3450-2019) [29], a BM-V pendulum friction tester was used to measure the British pendulum number (BPN) of different areas and perform temperature correction. The calculation formula is expressed as Equation (2) [30].

(2) $$BPN_{25}=BPN_{t}+\Delta_{BPN}$$

*BPN*_25_ denotes the pendulum value at the standard temperature of 25 °C, *BPN*_t_ represents the pendulum value measured at temperature t °C, and Δ*_BPN_* is the temperature correction value, as referenced in Table 2.

#### 2.2.3. Pull-Out Test

Based on the Technical Specifications for Maintenance of Cement Concrete Pavement in Civil Aerodrome (MH/T 5084-2025) [31], test blocks were molded using a tri-steel mold with dimensions of 40 mm × 40 mm × 160 mm. After molding, the blocks were cut into reference blocks with dimensions of 40 mm × 40 mm × 10 mm to meet the bonding performance testing requirements. Three coating thicknesses of 1 mm, 3 mm, and 6 mm were designed. After curing for 6 h and 24 h, the pull-out strength was tested using an LBY-VI bond strength tester, with a test temperature of 20 °C and a speed of 5 mm/min.

#### 2.2.4. Bonding Flexural Test

**Figure 4:**
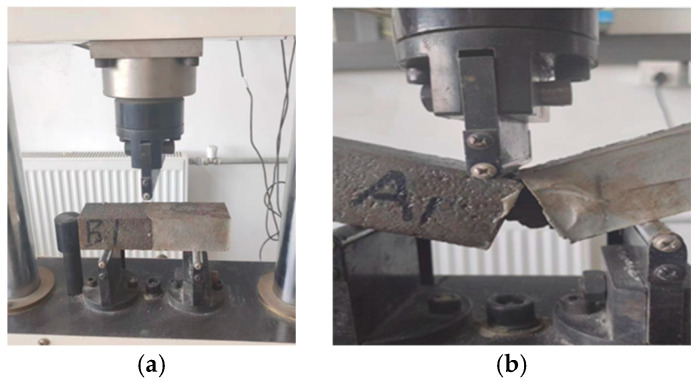
Method for determining bond flexural test: (**a**) Sample installation; (**b**) Load completion.

The bond flexural test was conducted according to the Testing Methods of Cement and Concrete for Highway Engineering (JTG 3420-2020) [32]. The triple-gang steel mold-formed specimens were cut into 40 mm × 40 mm × 80 mm concrete blocks, which were then filled with the test coating, vibrated on a shaking table, smoothed with a steel trowel, and finally covered with plastic film for molding. The two movable supports were adjusted to ensure the specimen was placed horizontally on the support, with its molded surface facing upward. In accordance with the concrete properties exhibited in this experiment, a loading rate of 0.05 MPa/s was applied until the specimen reached failure. The ultimate load at failure, the maximum load, and the location of fracture at the bottom edge of the specimen were recorded. The flexural bond strength between the repair material and concrete can be calculated using Equation (3). The test scenario is shown in Figure 4.

(3) $$R_{f}=\frac{\left(1.5\times F\times L\right)}{(b\times h^{2})}$$

*R_f_* is the flexural bonding strength between the repair material and the concrete, in MPa; *F* is the load pressure measured by the flexural testing machine, in N; *L* is the center-to-center distance of the lower support cylinders, with the standard fixture value *L* = 100 mm; b is the width of the specimen cross-section, i.e., 40 mm; and *h* is the height of the specimen cross-section, i.e., 40 mm.

#### 2.2.5. Freeze–Thaw Cycle Test

The freeze–thaw cycle test was carried out using the rapid freezing method specified in the Test Methods of Cement and Cement Concrete for Highway Engineering (JTG 3420-2020) [32]. To meet the requirements for frost resistance testing, standard prism specimens with dimensions of 100 mm × 100 mm × 400 mm were prepared. The TYC-HDK model rapid freezing-thawing test machine was used to conduct the freezing-thawing cycles for different numbers of cycles. During the test, the central temperature of the specimens was controlled at −28 °C and 5 °C, while the antifreeze solution temperature was kept at −45 °C. The mass loss of the specimens before and after the test was measured.

## 3. Results and Discussion

### 3.1. Identification and Evaluation of Surface Texture Characteristics of Coatings

#### 3.1.1. Mean Profile Depth

**Figure 5:**
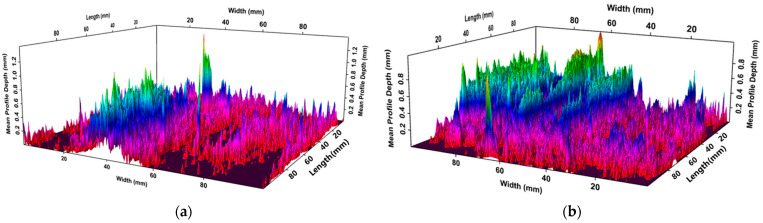
MPD visualized 3D topography: (**a**) No coating; (**b**) P1 coating.

The MPD is commonly used for texture structure characterization. The visual 3D topography of the MPD was obtained using Ames Texture Scanner professional software (version 3.0), as shown in Figure 5. The MPD was calculated for each coating type to provide a quantitative assessment of differences in microtexture.

**Figure 6:**
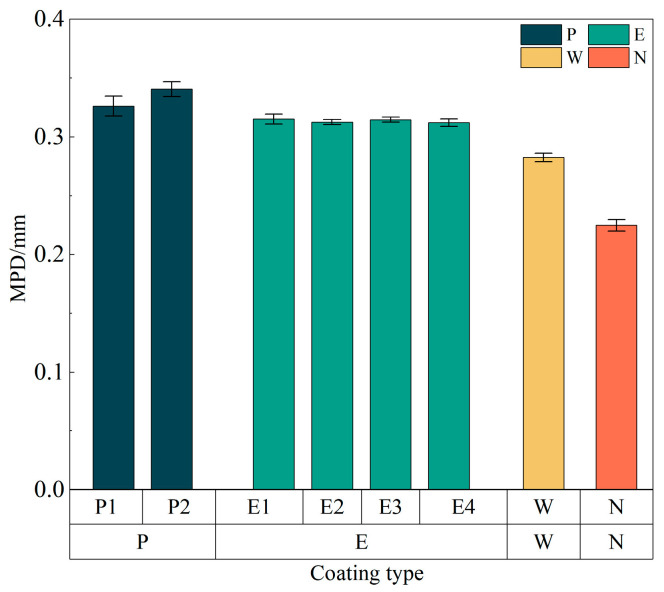
MPD values of different formulation coatings.

The visual 3D topography of the coatings is represented using a red-blue-green gradient, where red indicates the minimum MPD and green represents the maximum. Compared with the uncoated specimen, the coated samples exhibit a notable expansion of regions corresponding to higher MPD values, indicating an overall increase in surface microtexture. As demonstrated in Figure 6, the mean MPD values for the P, E, and W coatings increased by 43.4%, 34.7%, and 21.6%, respectively, relative to the uncoated control. Although the liquid components differed among the coatings, the microtexture distributions on the coated surfaces displayed substantial variations. Nevertheless, the consistent increase in MPD across all coatings demonstrates the significant effectiveness of the nanocoating materials in enhancing pavement skid resistance.

#### 3.1.2. Slope Spectral Density

**Figure 7:**
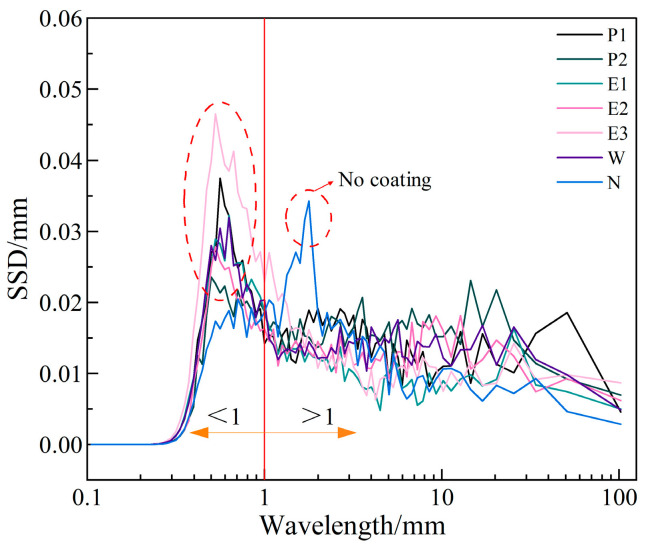
SSD distribution curves of different formulation coatings.

In terms of surface texture parameters for characterizing skid resistance, it is generally accepted that texture wavelengths below 1 mm are positively correlated with skid performance, with a correlation exceeding 80% [33]. Concurrently, the SSD provides a more accurate representation of surface texture wavelengths, and the peak SSD value corresponding to a wavelength can be regarded as the characteristic wavelength of the constructed texture within the scanned area [28]. Accordingly, the SSD distribution curves for each coating formulation were plotted in Figure 7 to quantitatively assess the microtextural characteristics of the coatings.

From Figure 7, the SSD peak value of the uncoated concrete surface appears in texture wavelengths greater than 1 mm, whereas after coating, the SSD peak value concentrates around 0.5 mm. The reason for this is that the coating material forms multiple micro-protrusions on the surface of the concrete matrix, enriching the surface microtexture and creating an effect like forward texture. This finding provides a microscopic explanation for how coating materials improve the anti-slip performance of concrete surfaces. Some studies have pointed out [34] that forward texture construction has better anti-slip effects than negative texture. Especially for wet and slippery surfaces, the adhesion mechanism arises from the bonding force between the tire and the surface micro-protrusions, and a coarser texture construction can break through the thin water layer on the surface, promoting contact between the road surface and the tire. This results in frictional attraction to enhance anti-slip performance [35]. In conclusion, the improvement effect of coating materials on anti-slip performance is particularly significant. Comparing different coating formulations, if only considering anti-slip performance improvement, P or E coatings are recommended. It should be noted that the concrete surface before coating is roughened, which is equivalent to the surface of a newly built cement road, and it already has a good anti-slip texture. If a worn road surface sample is used as the baseline, the improvement effect of the coating materials is expected to be even more significant.

### 3.2. Evaluation of Skid Resistance Based on BPN

**Figure 8:**
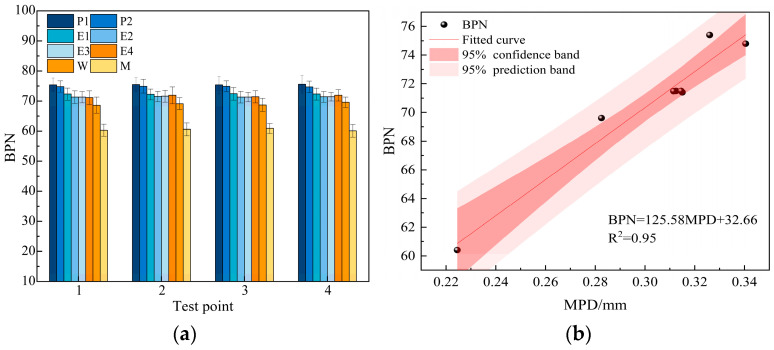
BPN and its correlation with MPD of coatings: (**a**) BPN characterization results; (**b**) BPN and MPD correlation.

To evaluate the anti-slip performance of the coating materials, different coatings were applied to concrete reference plates prepared in the laboratory, and a British pendulum test was conducted. The BPN results for each coating type are shown in Figure 8a. The correlation between MPD and BPN was also calculated, with the results presented in Figure 8b.

As demonstrated in Figure 8a, the uncoated concrete surface exhibited BPN values primarily around 60. After application of the coatings, the BPN for P, E, and W coatings were found to be concentrated around 75, 72, and 69, respectively, representing improvement rates of approximately 25%, 20%, and 15%. This indicates that the application of coatings can substantially augment the slip resistance of concrete surfaces. Comparative analysis suggests that the P coating achieves the highest BPN, followed by the E coating, while the W coating exhibits the lowest. This phenomenon is attributed to the styrene-butadiene emulsion, which functions as the liquid component of the P coating, achieving optimal integration with the solid powder. This, in turn, results in an increase in the micro-roughness of the material surface. As demonstrated in Figure 8b, MPD demonstrates a highly significant correlation with BPN, with a fitted correlation coefficient (R^2^) of 0.95, meaning that 95% of the variance in BPN is explained by MPD. A significance test (*t*-test) confirmed that this correlation is statistically significant with a *p*-value of less than 0.001 (*p* < 0.001). The narrow confidence interval indicates a high degree of fitting accuracy, thus confirming the consistency of test results across different scales.

When comparing these results with existing international literature, the exceptional skid resistance of these coatings becomes even more apparent. A study by Arabzadeh et al. on superhydrophobic coatings for concrete pavements found that applying a PTFE/PEEK coating, despite its water-repellent benefits, resulted in a reduction in BPN, ranging from 32% to 41% [36]. In stark contrast, the solid–liquid two-component coatings developed in this study consistently enhanced the BPN by 15% to 25%. This fundamental difference underscores the effectiveness of our coating strategy in achieving the primary goal of rapid texture reconstruction, significantly improving the critical skid resistance property.

### 3.3. Evaluation Coating–Concrete Interface Performance

#### 3.3.1. Interface Pull-Out Strength

**Figure 9:**
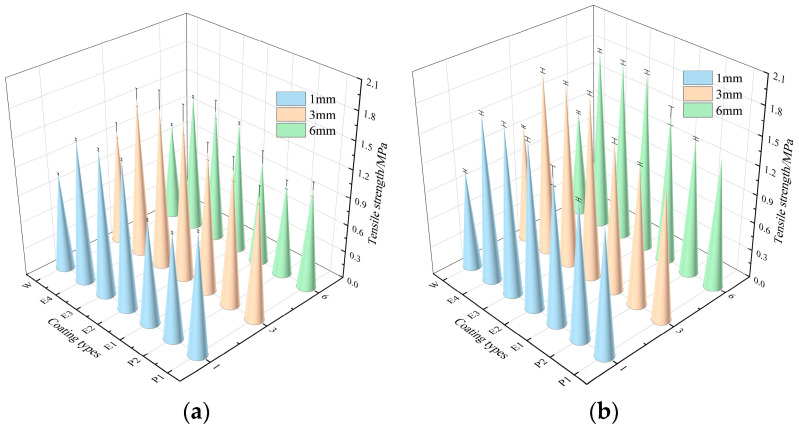
Pull-out test results at different curing times: (**a**) 6 h; (**b**) 24 h.

To characterize the interfacial bonding performance between the coating and the concrete substrate and to investigate the effect of coating thickness on bonding, three coating thicknesses (1 mm, 3 mm, and 6 mm) were designed. After curing for 6 and 24 h, pull-out strength tests were performed, and the results are presented in Figure 9.

From Figure 9, both the type of liquid component and the mixing ratio affect the interfacial bonding performance. Moreover, as the proportion of the liquid component decreases and the proportion of the solid component increases, the pull-out strength improves, indicating better interfacial bonding performance. Among the coatings, the E coatings (E2, E3, E4) have the highest pull-out strength, significantly higher than that of the P and W coatings. For a coating thickness of 3 mm, the maximum pull-out strengths for the P, E and W coatings after curing for 6 h are 1.46 MPa (P2), 1.71 MPa (E3), and 1.25 MPa (W), respectively; after curing for 24 h, the strengths are 1.47 MPa (P2), 1.91 MPa (E4), and 1.24 MPa (W). It is evident that the E coating has the advantage of fast curing and high strength. This is because waterborne epoxy resin reacts with the curing agent to form a physical-chemical crosslinked structure, which enhances the cohesion of the binder and its adhesion to the substrate. The cured epoxy resin also has higher cohesive strength, which helps to improve its bonding performance with the substrate [37].

Additionally, with an increase in coating thickness, the pull-out strength first increases and then decreases. When the coating thickness is 3 mm, the pull-out strength reaches its maximum. For a smaller thickness, small particles on the substrate surface protrude beyond the coating thickness, leading to a local lack of material and lower pull-out strength. When the coating thickness is 3 mm, more of the coating fills the uneven areas and gaps at the interface, effectively filling the existing voids and increasing the contact area between the coating and the concrete, thus improving the pull-out strength. For thicker coatings, interlayer slip within the coating may occur, reducing the pull-out strength. Therefore, it is recommended to control the optimal coating thickness in the range of 2–4 mm. According to the existing studies [38], high-performance coatings have a bonding strength of about 1.2 MPa after curing for 24 h. In this study, the W coating exhibited pull-out strengths between 1.10 and 1.35 MPa, while the other coatings all exceeded 1.2 MPa, demonstrating that the interfacial bonding performance of the developed coatings is excellent and comparable to, or even surpasses, that of existing commercial coatings.

#### 3.3.2. Flexural Bond Strength

**Figure 10:**
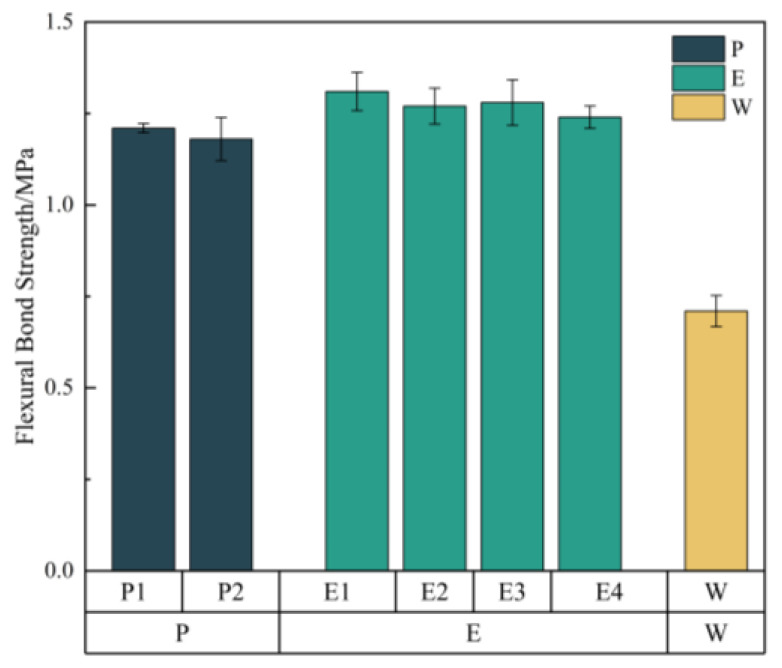
The bonding flexural strength test results of different coatings.

Each coating formulation was tested five times, and the bonding flexural strength was calculated as the average of the measurements. The results are presented in Figure 10.

For all specimens, fractures occurred at the interface between the repair material and the substrate. As demonstrated in Figure 10, the bonding flexural strength of the E1 coating is the highest, at 1.31 MPa. This phenomenon is likely attributable to substantial alterations in the microstructure that occur during the incorporation of the aggregate and filler into the epoxy resin. The aggregate and filler are bonded together by a thin film of epoxy. A discernible network structure is observed in the lower parts and the areas surrounding the needle-like and flake-like particles. These films form a bridge across the pores, thereby reducing the pore volume, and the epoxy resin intertwines and forms a network structure [39]. During the curing process of the epoxy mortar, the epoxy resin permeates and forms a film between the aggregate and filler, thereby increasing the mortar’s density and strength of bond. This results in enhanced mechanical properties and increased impermeability, which explains why the bonding flexural strength of the E coating is the highest. Furthermore, the P coating demonstrates comparable performance, while the W coating exhibits the lowest recorded strength.

### 3.4. Evaluation of Freeze Resistance

**Figure 11:**
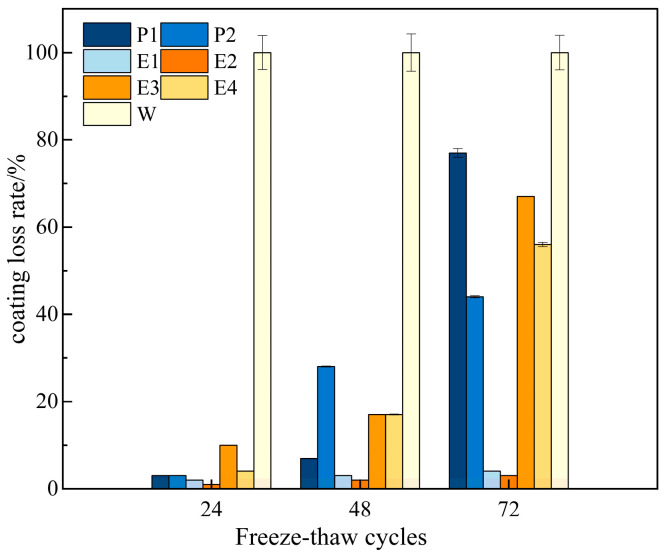
Characterization results of freeze–thaw tests for different coatings.

The rapid freeze–thaw test is employed to evaluate the protective performance of coating materials on concrete specimens. To ensure the accuracy of the test, the sides of the specimens are sealed with epoxy adhesive, while the top and bottom surfaces are coated with the material under investigation. To quantify the protective effect of the coating, the loss of the coating is tested after every 24 freeze–thaw cycles. The test results for each coating are shown in Figure 11.

As shown in Figure 11, the coating loss rate increases progressively with the number of freeze–thaw cycles. Among all coatings, the W coating exhibits the highest loss rate, experiencing complete detachment after 24 cycles. In contrast, the E coating (E2) demonstrates the lowest loss rate at 3%, showing a 97% improvement compared to the W coating. The loss rates for E1, E3, and E4 are 4%, 67%, and 56%, respectively. The P coating (P1 and P2) exhibits loss rates of 77% and 44%, respectively. These results indicate that both E and P coatings significantly enhance freeze–thaw resistance. This improvement can be attributed to the nanoparticles contained in the coatings, which provide a micro-aggregate filling effect that effectively fills the tiny pores within the concrete, thereby reducing the internal expansion stress caused by freeze–thaw cycles. The E coating (E2 and E1) offers the best protection, as the waterborne epoxy resin densifies the cement structure, enhancing its impermeability [40]. In summary, from a freeze–thaw protection perspective, the E coating is recommended as the priority choice. However, the P coating can also be considered as an alternative when cost control is a factor. Furthermore, regarding freeze–thaw durability, a study by Guo and Weng [6] concluded that conventional epoxy resin exhibited the poorest performance among several surface treatments. In stark contrast, our waterborne epoxy coating (E) demonstrated the best freeze–thaw resistance.

## 4. Conclusions

This study investigates the formulation design and performance evaluation of composite coating materials for application on airport cement pavements. Seven solid–liquid, two-component coating formulations were developed, incorporating three types of coatings: emulsion coating (P), waterborne epoxy coating (E), and water-based coating (W). The performance of these coatings was comprehensively assessed, and appropriate formulations were recommended based on various repair needs. The main conclusions are as follows:

(1) The textural characterization analysis reveals that, in comparison to the uncoated specimens, the mean profile depth (MPD) of the P, E, and W coatings increased by 43.4%, 34.7%, and 21.6%, respectively. Additionally, the peak band of the Slope Spectral Density (SSD) on the concrete surface shifted from a value greater than 1 mm to approximately 0.5 mm before and after the application of the coatings.
(2) Laboratory test results reveal that, in comparison to the uncoated sample, the British Pendulum Number (BPN) of the P, E, and W coatings increased by approximately 25%, 20%, and 15%, respectively. The analysis identified a significant correlation between the mean profile depth (MPD) and BPN (R^2^ = 0.95). This demonstrates that the coating surface texture exhibits superior performance, thereby elucidating the mechanism of enhanced slip resistance from a textural perspective.
(3) The results of the pull-out test indicate that, when the coating thickness is 3 mm and the curing time is either 6 h or 24 h, the E coating exhibits the maximum pull-out strength. As the coating thickness increases, the pull-out strength follows a pattern of initial increase followed by a decrease. It is recommended that the coating thickness be maintained within the range of 2–4 mm. The results of the flexural strength test show that the E coating exhibited the highest bond flexural strength, followed by the P coating, while the W coating showed the lowest performance.
(4) The results of the freeze–thaw test indicate that after freezing and thawing, the W coating exhibited the highest loss rate, while the E coating demonstrated the lowest loss rate. From the perspective of freezer protection, the E coating is the preferred choice. However, for cost control purposes, the P coating may also be considered.
(5) The findings of this study underscore the potential of the newly developed coating technology in significantly improving the skid resistance and frost resistance of cement pavement, particularly in the context of airport runways. Beyond its immediate benefits, this innovation offers a promising approach for rapid pavement maintenance, minimizing operational disruptions. While the laboratory-based results provide a strong foundation for future applications, additional research is needed to evaluate the coating’s performance under real-world conditions. A key direction for future work will involve field validation and long-term durability testing. These efforts will be crucial in bridging the gap between laboratory-scale innovations and their full-scale engineering implementation, ensuring the coating’s effectiveness and viability in actual pavement maintenance scenarios.

## Figures and Tables

**Table 1:** Coating formulations and labeling.

Type	Code	Solid–Liquid Ratio	Liquid Component Ratio	Fluidity/mm
Self-prepared emulsions (P)	P1	2:1	Water: Emulsion = 1:1	110
	P2	10:4.5	Water: Emulsion = 1:1	90
Waterborne epoxy (E)	E1	2:1	Water: Epoxy = 1.5:1	120
	E2	5:2	Water: Epoxy = 2:1	116
	E3	3:1	Water: Epoxy = 3:1	100
	E4	3:1	Water: Epoxy = 2:1	92
Water(W)	W	3:1	water	130

**Table 2:** Temperature correction value [29].

Temperature	0	5	10	15	20	25	30	35	40
Δ_BPN_	−6	−4	−3	−2	0	2	3	5	7

## Data Availability

The original contributions presented in the study are included in the article, further inquiries can be directed to the corresponding author.
